# The House of Lords and Nurse Registration

**Published:** 1919-06-07

**Authors:** 


### THE MATRONS' AND SISTERS' DEPARTMENT

### THE HOUSE OF LORDS AND NURSE REGISTRATION.

THE animated debate on the occasion of the second reading of the Registration Bill in the House of Lords on May 27 showed far more real appreciation of the facts at issue than that which heralded the Central Committee's Bill in the House of Commons. Members of the House of Commons, notwithstanding their sympathetic attitude towards nurses who desire to organise their own profession, have shown strange reluctance to go to the root of the matter. Yet there is nothing in the least technical or difficult to understand in the controversy about the two Bills. It merely requires a little patient investigation of the facts such as lies within the power of a plain man to devote to the subject. The Standing Committee of the House of Commons entirely neglected to examine the facts. And hence the supposed agreement at which they arrived was in effect worthless.

Here on the one side are certain societies which profess to represent the nurses, the genuine workers, the rank and file. Is it unreasonable to ask how many of the nurses they represent? In the Central Committee's Bill the promoters state that it is backed by 30,000 persons. But this assertion is justified only by the inclusion of the entire British Medical Association. What is the membership of this Association? We are informed on good authority that it does not come short of 23,000 persons, and this number who are certainly not nurses ought to be deducted. This reduces the numbers for the "independent nursing societies" to 7,000, and of this number some 2,500 are registered as trained in Fever Nursing. Deduct these and there remain 4,500 who may or may not be fully-trained nurses. But how can the total membership of these societies be arrived at as an audited figure? In truth these societies melt into one another. Membership of one seems to form a passport to membership of another. Certain people seem to belong to the whole seven. Ought they to be counted seven times over? Are they so counted in order to reach with the entire British Medical Association the imposing total of 30,000? These things are ascertainable. There is no reason for secrecy or mystery about the strength of membership in a society, for there is nothing necessarily to be ashamed of in having only a small following. A band of a few ardent and convinced reformers may effect great reforms. But no one effects reforms by pretending to a large following which does not exist. The Central Committee lay great stress on the importance of numbers and have made definite statements about the numerical strength of their following. They maintain that the Bill which they promote is desired by the nursing profession. Were this assertion correct it could be demonstrated in half an hour. But the Central Committee refuse to substantiate it. The House of Commons while lending itself with exuberance to an elaborate debate on nurses and nursing did not through its Standing Committee make the slightest attempt to ascertain the number of nurses who are in favour of the Central Committee's Bill. It is due to nurses that in matters affecting their interests for generations to come assertions should not be taken for granted.

On the other hand we find nurses have displayed an enthusiasm without parallel in the history of their profession to enrol themselves in the self-governing body known as the College of Nursing. Members of Parliament seldom lack some experience in the art of attracting support for societies. How many members at a guinea membership fee could be attracted even for an association of great national interest merely by widespread advertisement and influential backing? We should say two or at most three thousand. And either figure would mean very adroit publicity. But consider the case of the College of Nursing. Within three years its membership has reached 14,000, and every day the applications come in faster and faster. Few of these women have an income exceeding two hundred a year. Yet they cheerfully find a guinea because of all that the College means to them. These fourteen thousand have come in voluntarily from every part of these islands. They belong to every branch of nursing. They are of every rank from matrons to recently certificated nurses. How preposterously silly to assert, as Lord Dufferin dared to do in the House of Lords, that they have joined the College in order that they may be registered without examination and at a lower fee! The College is the most wonderfully representative and enthusiastic body which has ever in any profession risen to a sense of its opportunities and great future.

Is it possible that Parliament will pass over this great body of professional women as unworthy of a hearing, and hand over the government of their profession to persons wholly out of touch with their aspirations? The College has at least earned the right to demand that the facts be impartially examined.

